# Olive Crown Porosity Measurement Based on Radiation Transmittance: An Assessment of Pruning Effect

**DOI:** 10.3390/s16050723

**Published:** 2016-05-19

**Authors:** Francisco J. Castillo-Ruiz, Sergio Castro-Garcia, Gregorio L. Blanco-Roldan, Rafael R. Sola-Guirado, Jesus A. Gil-Ribes

**Affiliations:** Department of rural engineering, University of Córdoba, Campus de Rabanales, Córdoba 14071, Spain; scastro@uco.es (S.C.-G.); ir3blrog@uco.es (G.L.B.-R.); ruben.sola.trabajo@gmail.com (R.R.S.-G.); gilribes@uco.es (J.A.G.-R.)

**Keywords:** *Olea europaea* L., leaf area density, leaf area index, gap fraction, radiation balance, trunk shaker, canopy shaker, mechanical pruning, harvesting

## Abstract

Crown porosity influences radiation interception, air movement through the fruit orchard, spray penetration, and harvesting operation in fruit crops. The aim of the present study was to develop an accurate and reliable methodology based on transmitted radiation measurements to assess the porosity of traditional olive trees under different pruning treatments. Transmitted radiation was employed as an indirect method to measure crown porosity in two olive orchards of the Picual and Hojiblanca cultivars. Additionally, three different pruning treatments were considered to determine if the pruning system influences crown porosity. This study evaluated the accuracy and repeatability of four algorithms in measuring crown porosity under different solar zenith angles. From a 14° to 30° solar zenith angle, the selected algorithm produced an absolute error of less than 5% and a repeatability higher than 0.9. The described method and selected algorithm proved satisfactory in field results, making it possible to measure crown porosity at different solar zenith angles. However, pruning fresh weight did not show any relationship with crown porosity due to the great differences between removed branches. A robust and accurate algorithm was selected for crown porosity measurements in traditional olive trees, making it possible to discern between different pruning treatments.

## 1. Introduction

The European Union is the world’s foremost olive oil and table olive producer with an average production of 2034.3 Gg of olive oil and 776.8 Gg of table olives in the last 6 years. Spain is the largest grower of olives; its production reached 1274.9 Gg of olive oil and 538.8 Gg of table olives in the same period [1]. In this country, the olive sector has a notable social and economic importance generating an average €1,886 million per year (from 2007 to 2012) and an estimated 46 million working days each year [2].

Because of this sector’s importance, a public pre-commercial procurement project, Mecaolivar, was set up to stimulate competitiveness and modernization in the olive sector through the introduction of innovations in olive machinery accompanied by an adaptation of trees through pruning [3].

Crown parameters are important in woody crops because they determine radiation extinction through canopy, and intercepted photosynthetically active radiation (PAR) determines important parameters such as fruit set, fruit density, and oil content [4]. Taking measurements of crown parameters in woody crops and forests is a difficult task that requires a high level of accuracy and represents a major effort in data post-processing. Tree canopies follow irregular shapes, although some features may be explained by mathematical models or coefficients. Canopy structure, leaf distribution, and crown porosity are important factors that determine crop yield and biomass production.

Several technologies are available to characterize tree crowns and forest architecture: radiation measurements [5], terrestrial canopy photography [6], terrestrial laser [7], Unmanned Aerial System (UAS) imagery [8], and lateral imagery analysis [9]. Furthermore, flowering assessment can be performed by using a smartphone application based on imagery analysis [10]. Imagery analysis is a highly accurate, cost-effective method for taking canopy measurements, but it presents serious difficulties when attempting to generate foliage to non-foliage pixel segmentation, mainly in crown shaded areas. This limitation can also be overtaken using upward photography. Therefore, imagery analysis is a highly accurate and reliable method when pixel segmentation is feasible; otherwise, alternative methods should be used. Radiation measurement is employed as an indirect method for canopy porosity measurement along the sunbeam direction.

Gap size distribution within canopy, or crown porosity, can be used to describe radiation penetration through tree structure as a function of zenith angle [11]. In olive orchards, very few studies have investigated canopy structure influence on harvesting operation and light penetration [12], while orchard layout [13,14] or crown porosity [15] have an important influence on yield. Radiation interception and its relation with olive productivity have been well-assessed, [16]. Daily intercepted PAR in high-density and super-high-density olive orchards varied from 6 to 25 mol/m^2^, representing between 15% and 60% of horizontal incident radiation [15]. However, other effects such as air movement through an olive orchard to reduce pests or disease outbreaks and spray penetration effectiveness have not yet been studied [17]. Additionally, porosity may influence canopy shaker efficiency during the harvesting process considering that canopy shakers may be more efficient in dense canopies. Woody parts in the tree canopy also affect PAR interception, air movement, and spray penetration. Moreover, some authors have recorded that wood area density affects PAR transmittance through the tree canopy, reducing PAR transmittance by 29% [18], although this effect has more importance in deciduous trees than in evergreens.

Pruning is a key factor in crown porosity adjustment, mainly for traditional olive orchards. Traditionally, pruning has been used as a cultural practice to regulate production levels and helps to avoid pests and disease in rain-fed olive groves [19]. Garcıa-Verdugo *et al*. [20] found no significant differences in incident irradiance for wild olives in similar topographic conditions either by direct measurement or by estimation from hemispherical photographs. In some windy areas, pruning can also act as a preventive practice to protect trees from being blown down, considering that it has been demonstrated that pruning affects tree performance against hurricane force winds [21].

How pruning affects olive crown porosity has not been quantified. Therefore, evaluation of the influence of pruning on olive canopy structure is an important issue that could affect crown porosity, radiation transmittance, clumping coefficient, and other geometrical features or coefficients [19]. An assessment of how pruning regulates crown structure should be used to modify radiation balance in order to maximize crop production and machinery performance.

The objective of the present study is to develop an accurate and reliable methodology, based on transmitted radiation measurements, to assess the porosity of traditional olive trees under different pruning treatments. The influence of direct and diffuse PAR on crown porosity and data processing is also evaluated.

## 2. Materials and Methods

### 2.1. Pruning Treatments

Pruning was performed in two traditional multi-trunk olive (*Olea europaea* L.) orchards in Cordoba (37.717° N, 4.806° W) and Jaen (37.738° N, 4.145° W) with Hojiblanca and Picual cultivars, respectively. Those cultivars had been selected because they represent more than 43% of the total olive growing surface in Spain [22], and they show greater importance in multi trunk traditional olive orchards. Trees were over 100 years old and in good health and phytosanitary condition, and tree density varied from 70 to 80 trees ha^−1^.

Two pruning treatments were applied to adapt tree architecture to two different harvesting methods: trunk shakers [23] and canopy shakers [24]. Finally, a mechanical pruning treatment was performed that focused on cost saving. The influence of these pruning systems on tree crown architecture and porosity was recorded.

A pruning schedule was performed according to Table 1 in spring from 2013 to 2015. Fresh weight was composed of wood, buds, and leaves. It was obtained separately from each tree and in each pruning year. Pruning frequency was established in accordance with the pruning system’s purpose. Therefore, the trunk-shaker-targeted pruning was applied every two years, while canopy-shaker-targeted pruning, which required more intense adaption, was performed annually. Mechanical pruning was applied only once because a large canopy volume was previously needed to get adequate results. Pruning was carried out two or three months before PAR measures to include the budding effect. Both orchards started the pruning program in 2013, and it continues to date.

Pruning treatment features are described below: 

Trunk-shaker-targeted pruning: This pruning system is widely used in Southern Spain as the standard pruning method in many olive orchards. For this study, a hand-held chainsaw was used to perform the cuts. Lower branches that hindered trunk shaker driver vision were removed, together with the inner branches, which were more difficult to reach with pole manual harvesting or hand-held devices. Pole manual harvesting or shaker combs were used to aid the trunk shaker in order to reach a harvesting efficiency of over 85% [25]. Renewal pruning was also used for scaffolds with the aim of maintaining an adequate yield and tree vigor while keeping a high leaf/wood ratio. This tree architecture made it possible to achieve high harvesting efficiency, high olive yield, and adequate effective field capacity during the harvesting operation with trunk shakers.

Canopy-shaker-targeted pruning: This pruning system was developed with the aim of adapting the tree structure in order to obtain higher efficiency and canopy shaker performance in traditional olive trees with several trunks [24] or in large-sized canopies [26]. A hand-held chainsaw was used to perform the cuts. Inner branches were removed because canopy shaker sticks could not reach the canopy central volume while outer bearing branches were kept. Outer branches that hindered continuous canopy shaker work around the tree canopy were also removed to procure a round canopy perimeter. Renewal pruning was only used for secondary limbs when it enhanced a round path around the tree canopy.

Mechanical pruning: Pruning here was performed by a tractor-mounted circular saw pruner, which was hitched to a frontal loader. Light inner shoot clearing was performed with a hand-held chainsaw, which was used to clean up inappropriate mechanical cuts or wood without leaves. This pruning system aimed to increase effective field capacity in the pruning operation and to reduce pruning costs.

### 2.2. Method for Ceptometer Calibration

An SS1 Sunscan ceptometer (Delta-T devices, Cambridge, UK) was used to measure PAR under olive canopies. This device has a measurement range between 0 and 2500 μmol·m^−2^·s^−1^ and was connected to a PC through a serial port to gather data. The radiometer probe has 64 individual photodiode readers, which provided 64 individual readings. The mean value was obtained from every probe position to determine the radiation that passed through the porous sheet or the tree canopy.

**Figure 1:**
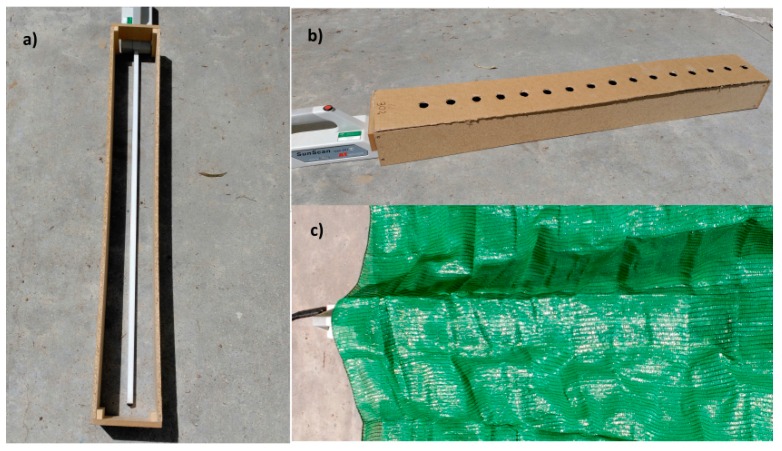
Ceptometer during calibration process. (**a**) Ceptometer with wood structure to avoid diffuse radiation at sun-exposed conditions. (**b**) Ceptometer with wood structure under drilled sheets. (**c**) Ceptometer with wood structure under porous nets.

The ceptometer was calibrated under sun radiation. A wood structure was used to avoid diffuse radiation reaching the probe and affecting the final value. Firstly, PAR transmittance to porosity was modeled by using drilled sheets placed over the radiometer (Figure 1). There were different sheets with a different number of holes, but a constant distance between holes was always kept, taking the center of the sheet as a reference point. The dimension of the drilled sheets was 150 × 1000 mm (width × length), and the holes had a 24-mm diameter.

Secondly, another method was used to assess how the solar zenith angle affected PAR transmittance under porous media. Porous nets were used to evaluate accuracy and repeatability of transmitted PAR measurements (Figure 1). The same wood structure was used to avoid diffuse radiation, but nets with known porosity were placed over the probe, and measurements were taken over a large range of solar zenith angles from 14° to 56°.

### 2.3. In-Field Measurements of Transmitted Radiation

In-field measurements were taken under pruned traditional olive trees, placing the probe at ground level on the sides of a 1-m sided grid, spread out under the canopy Only those sides that were within the shadow of the olive crown were considered. Afterwards, PAR was calculated as a mean value of the measurements to obtain a mean value of olive crown porosity. Sun-exposed PAR and completely shaded PAR were measured for each tree. Ten trees of each pruning treatment were measured per year and per cultivar to assess how crown porosity changed depending on pruning treatment.

### 2.4. Algorithms for Data Processing

Crown porosity was calculated using under-crown PAR (PAR_Under crown_), sun-exposed PAR as direct radiation (PAR_Sun_), and completely shaded PAR as diffuse radiation (PAR_Shaded_). Diffuse radiation was measured placing the probe at ground level under a solid sheet 2 m wide and 2 m long, placed 0.4 m above the soil in a completely sun-exposed area.

Algorithm 1 used the drilled sheet experimental calibration method. The calibration method was performed taking measurements under sun-exposed conditions, keeping the same solar zenith angle and varying drilled sheet porosity. Eight measurements were taken at different sheet porosities and three repetitions were made for each porosity value. Equation (1) shows the calculated model (RMSE = 1.89; ρ < 0.01; R^2^ = 0.99).

(1) $$\phi_{1}=0.059\cdot {\text{PAR}}_{\text{Under crown}}+5.715$$

Algorithm 2 supposed that PAR and porosity are related by a linear regression, as is shown in Algorithm 1 (ρ < 0.01; R^2^ = 0.99). A hypothesized model intercepted the Y-axis at 0 value because the shaded PAR was not considered as diffuse radiation.

(2) $$\phi_{2}=\frac{{\text{PAR}}_{\text{Under crown}}}{{\text{PAR}}_{\text{Sun}}}\;100$$

Algorithm 3 considered diffuse radiation as shaded PAR, which influenced the calibration model and the under-crown measurements. Shaded PAR was considered as an unwanted steady offset, which was removed to zero the measure.

(3) $$\phi_{3}=\frac{{\text{PAR}}_{\text{Under crown}}-{\text{PAR}}_{\text{Shaded}}}{{\text{PAR}}_{\text{Sun}}-{\text{PAR}}_{\text{Shaded}}}\;100$$

Algorithm 4 introduced diffuse radiation as shaded PAR, but only in the calibration model. This algorithm assumes that the canopy is a complex solid net, where diffuse radiation represents an important fraction that cannot be subtracted to under-crown PAR. However, diffuse radiation was taken into account to determine calibration interval.

(4) $$\phi_{4}=\frac{{\text{PAR}}_{\text{Under crown}}}{{\text{PAR}}_{\text{Sun}}-{\text{PAR}}_{\text{Shaded}}}\;100$$

Algorithms 2–4 are based on an equation proposed in [27] that relates leaf area index (LAI) to PAR; these authors describe the importance of both the direct and the diffuse radiation fraction in determining crown porosity.

To evaluate the performance of in-field algorithms, 18 trees (6 per pruning treatment) were measured twice in the same cloudless day. They were first measured in the morning when the solar zenith angle was high, between 28° and 43°. Measurements were later taken under the tree crown around the noon, when the solar declination angle was low, between 14° and 18°. Porosity was calculated using the different suggested algorithms, and it was compared to determine how robust the method was against solar zenith angle variations.

### 2.5. Evaluation of Algorithm Accuracy and Repeatability

The accuracy and repeatability of measurements were also evaluated using porous nets as a known porous media at different solar zenith angles. The porosity of measured and known nets was compared to obtain absolute error values. Repeatability (r_Φ_) was also calculated using the variance between porosity measurements under all porous media, from 15% to 55%, at different solar zenith angles ($\sigma_{\theta }^{2}$), and using the variance between porosity measurements under each porous medium for different solar zenith angles ($\sigma_{\phi }^{2}$).

(5) $$r_{\phi }=\frac{\sigma_{\theta }^{2}}{\sigma_{\theta }^{2}+\sigma_{\phi }^{2}}$$

## 3. Results and Discussion

### 3.1. Ceptometer Calibration and Evaluation with Known Porous Media

**Figure 2:**
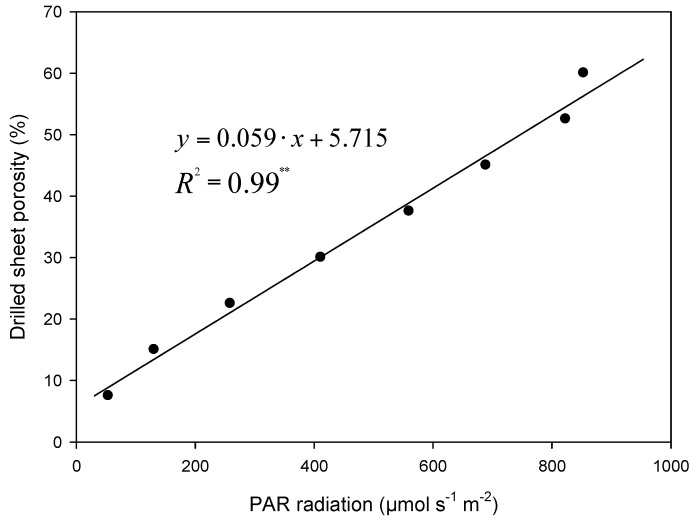
Sunscan calibration and regression between PAR and porosity using drilled sheets to shade the probe.

The calibration model using drilled sheets demonstrated that there was a porosity interval where porosity and PAR under a porous medium were related linearly (Figure 2). Porosities over 60% could not be tested due to geometrical constraints in the drilled sheet layout.

The drilled sheet calibration method was used to determine Algorithm 1. It imposed substantial or limited lighted or shaded areas (Figure 2). This calibration method generated a noticeable regular sunfleck pattern with shaded and sunlit areas, while real olive trees often show a highly variable spatial distribution [19]. This variability might be represented by a clumping coefficient, although it is more a fitting than a bio-physically meaningful parameter to adjust theoretical to real canopy volume [28]. In the canopy, leaf inclination, leaf reflectance, and transmittance also influence radiation interception by olives [29]; therefore, the shown calibration only demonstrated that there was a porosity interval at which transmitted PAR and porosity were linearly related within a porosity range from 7.5% to 60%.

**Figure 3:**
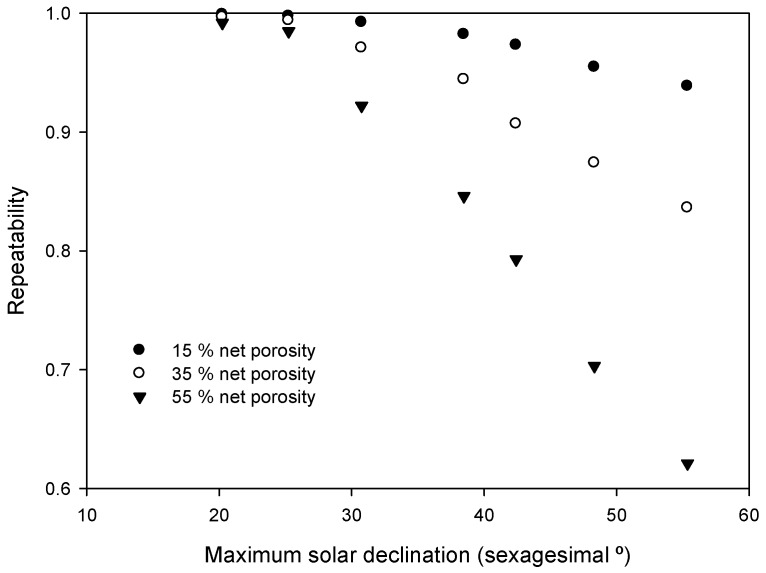
Repeatability depending on solar zenith angle and net porosity.

**Figure 4:**
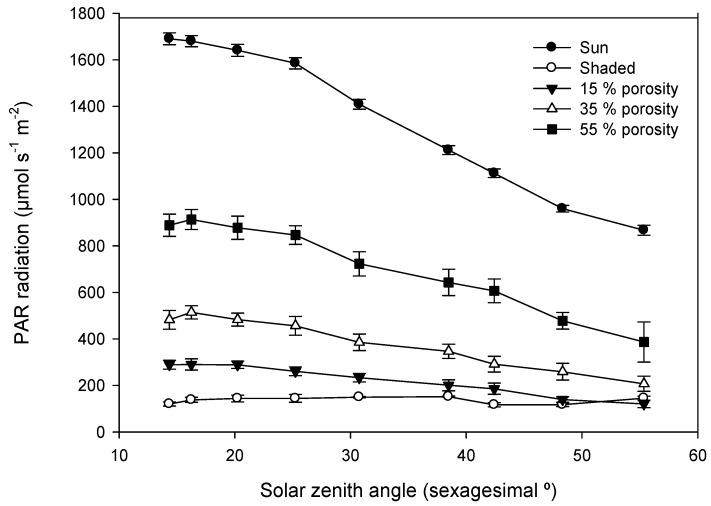
PAR measures evolution depending on solar zenith angle and radiation exposure under porous nets.

PAR under porous media was affected by solar zenith angle; therefore, there was a range of time in which PAR could be measured with an adequate repeatability. Measurements taken only below 31° solar zenith angle were considered to keep an adequate repeatability of over 0.9 within a porosity range from 15% to 55%. Repeatability of transmitted PAR at ground level was severely affected by solar zenith angle, and it also varied depending on medium porosity (Figure 3). For the tested range of porosities, differences in measured PAR displayed reduced differences when solar zenith angle was below 31° (Figure 4). It is stated in [30] that direct PAR within the crowns of wild olives does not vary during the day in summer conditions but does vary in winter conditions, while diffuse PAR does not differ during the day.

### 3.2. Assessment of Method and Algorithms Accuracy

**Figure 5:**
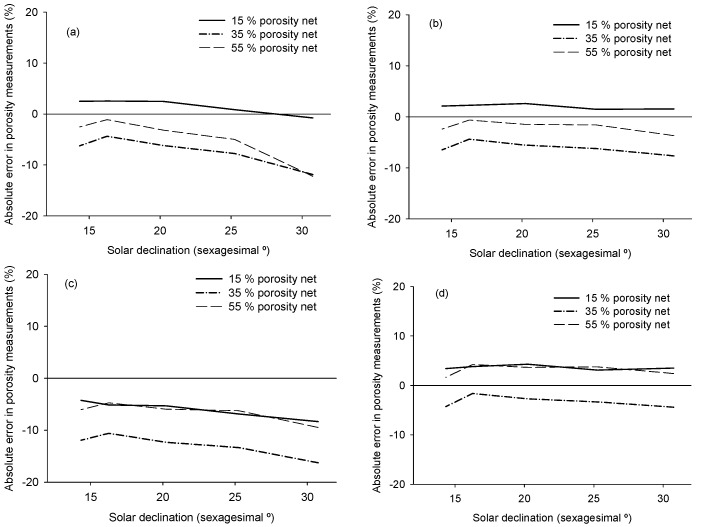
The accuracy of porosity measurement and absolute error of different porous nets depending on solar zenith angle. (**a**) Absolute error for Algorithm 1; (**b**) absolute error for algorithm 2; (**c**) absolute error for algorithm 3; (**d**) absolute error for algorithm 4.

The accuracy of porosity measurement varied depending on the calculation algorithm and solar zenith angle (Figure 5). On the one hand, the highest accuracy was provided by Algorithm 4 (absolute error < 5% crown porosity) without perceptible variations depending on solar zenith angle between 14° and 31°, while Algorithm 2 provided absolute errors up to 10% of crown porosity depending on solar zenith angle. On the other hand, Algorithms 1 and 3 produced low accuracy, and it varied depending on solar zenith angle (Figure 5). Instruments for indirect canopy measurements generally provide 20% accuracy, being mainly limited by the assumption of randomness [27]; thus, Algorithm 4 always produced an appropriate accuracy of over 95%. According the tested accuracy (Figure 5), the solar zenith angle range in which crown porosity can be measured, keeping accuracy over 95%, was below 30°. Moreover, within this solar zenith angle range, solar radiation displayed a similar slope between different porosities when radiation direction varied (Figure 4).

The accuracy of algorithms was evaluated by measuring PAR under porous nets and comparing the obtained results with known porosity. Algorithm 1 provided high accuracy when the solar zenith angle was low. However, this algorithm was highly affected by solar zenith angle. Algorithm 3 provided inaccurate and biased information, indicating that diffuse radiation represented an important fraction in PAR under porous nets, even at low solar zenith angles. Diversity within the canopy geometry also generates scattering radiation due to reflectance and transmittance, although leaf reflectance is much higher than leaf transmittance [31]. In addition, high zenith angles could modify the clumping coefficient that changes the canopy gap distribution [32], while low zenith angles provided more similar results to those that affect radiation direction transmittance. Therefore, it is advisable to measure the within canopy gap fraction at low solar zenith angles.

Finally, Algorithms 2 and 4 were valid in terms of accuracy to calculate olive crown porosity by means of PAR transmittance across the olive canopy due to their stability over a large range of solar zenith angles.

### 3.3. Ceptometer Evaluation for in-Field Conditions

The rigor of algorithms was evaluated by measuring tree crown porosity in the same trees at high and low solar zenith angles (Table 2). Algorithms 1 and 3 produced significant differences in crown porosity at different solar zenith angles according to the Wilcoxon test (ρ < 0.05). However, Algorithms 2 and 4 were suitable for measuring tree crown porosity at different solar zenith angles. Algorithm 4 produced better results at different incident directions of radiation. This algorithm used shaded PAR as diffuse radiation only to calibrate PAR in the measurement time range. Algorithm 4 demonstrated that olive crown porosity did not vary within a zenith direction from 14° to 43°. Thus, vertical crown porosity was equal at different zenith angles in this range. However, previous research has stated that olive canopy porosity or gap fraction varies depending on the point of view [17]. Thus, assessing the solar zenith angle interval at which crown porosity could be measured and the most robust algorithm to analyze data is an important issue to determine olive crown porosity accurately.

It was demonstrated that porous nets and olive canopy responded similarly to zenith angle, although canopy represented a 3D porous solid medium, while nets could be represented by a 2D solid porous medium. Algorithms 2 and 4 provided an adequate accuracy nevertheless. Table 2 displays a better behavior than Algorithm 4 for in-field performance, and it was demonstrated as the best algorithm to process PAR measurements for olive crown porosity calculation.

### 3.4. Pruning Influence on Crown Porosity

PAR measurements were useful to determine crown porosity, which changed depending on the pruning system (Table 3). Tree crown porosity changed depending on previous pruning, pests or diseases that affected the plant leaf area index. Mechanical pruning demonstrated significantly lower porosity values for two sampling dates than other pruning treatments. However, when mechanical pruning was conducted in 2014, crown porosity was lower than canopy-shaker-targeted pruning but higher than trunk-shaker-targeted pruning. This was due to the fact that trunk shaker pruning was not applied in this year. Olive pruning modifies crown porosity, which decreases when pruning was applied to every treatment. However, crown porosity did not correlate with pruning fresh weight in the same season, or with pruning cumulated fresh weight (Figure S1). This could be due to great differences in removed branches and the influence of wood weight, considering that branch fresh weight range went from 0.1 to more than 50 kg.

Crown porosity could also be affected by foliage sicknesses or tree structure. These facts explained why Hojiblanca cultivar provided lower crown porosity than Picual, which shows better resistance to foliage sicknesses [33] and more compact crown structure. Results demonstrate that the pruning system influences crown porosity, similar to previous research done in vines, demonstrating that orchard layout and training system are more important than cultivar or year for crown porosity [34]. In contrast, the year demonstrated to have a great influence on olive trees, mainly due to the fact that olive pruning is usually applied every two years, while vine pruning is applied every year. In olive trees, plant area density ranged from 1.16 to 2.72 m^2^·m^−3^, of which 91% consisted of leaf area [35]. Moreover, leaf area density shows values below 2 m^2^·m^−3^ [16], while values from 2.5 to 2.7 m^−2^·m^−3^ were reported in [36].

Pruning fresh weight did not show a clear trend considering pruning treatments and years (Table 4). This is due to pruning intensity varying depending on tree growth, canopy density and crown structure. On the one hand, in Hojiblanca cultivar, mechanical pruning and trunk-shaker-targeted pruning demonstrated similar fresh weight values, while canopy shaker pruning removes a higher weight of branches. On the other hand, in Picual cultivar, mechanical pruning demonstrated lower values of cumulated fresh weight, while trunk shaker and canopy shaker pruning residues depend on the year. Crown dimensions did not provide significant differences (ρ < 0.05) between pruning treatments except canopy volume measured for Hojiblanca cultivar in 2014 and skirt and tree heights measured for Picual cultivar (Table S1). Crown porosity was not influenced by crown dimensions in Hojiblanca cultivar, providing an inverse relationship between canopy volume and crown porosity in 2014. However, significant differences (ρ < 0.05) observed in Picual trees matched different tree and skirt heights, while canopy volume did not provide any results. Thus, further research is needed to clarify whether tree dimensions are related to crown porosity.

Radiation transmittance to ground level through the tree canopy was measured for 30° to 14° zenith angle transect, but are similar to horizontal porosity. It was measured for a super-high-density olive hedge at a medium height (1–1.5 m) with 15%, whereas, in the upper hedge section, porosity increases to 37% [36]. In high latitude locations, it has been described that crown porosity does not affect light measurements under tundra forests [37]. However, in lower latitudes, other authors state that 50% of the total irradiance was lost in the first 0.5–0.8 m within the olive tree canopy [30].

## 4. Conclusions

Taking olive crown porosity measurements using PAR transmittance through traditional olive trees crowns is a difficult task due to solar zenith angle variations. Direct and diffuse radiation was used to develop an accurate method to measure olive crown porosity by means of under-crown PAR measurements. Moreover, one of the tested algorithms provided an accurate and robust method that is robust over a range of different solar zenith angles, enabling a wide range of working conditions. Porosity and transmitted PAR regression did not provide suitable methods to determine olive crown porosity, but direct, diffuse, and transmitted PAR weighting allows for the processing of PAR data into crown porosity. Algorithm 4 used diffuse and direct PAR to determine crown porosity. This would be the most appropriate method to process PAR under the crown into porosity. The influence of diffuse radiation should not be dismissed when calculating under-crown PAR, but diffuse PAR should be deducted from sun-exposed PAR to obtain a reliable algorithm for data processing.

PAR measurements also made it possible to discern between different pruning treatments in traditional olive trees, making it an accurate and reliable method to evaluate crown porosity and canopy structure. Crown porosity in traditional olive orchards was related to the pruning system. Further research is needed to describe how pruning can affect crown porosity and intercepted radiation in the olive canopy, research that would also consider different training systems such as super-high-density, and high-density olive orchards.

## Figures and Tables

**Table 1:** Pruning schedule in both olive groves. X means the year in which pruning was applied for each treatment.

Pruning Treatment	2013	2014	2015
Trunk-shaker-targeted pruning	X		X
Canopy-shaker-targeted manual pruning	X	X	X
Mechanical manual pruning		X	

**Table 2:** Olive crown porosity (Φ) measurements depending on solar zenith angle. Values are mean ± standard deviation. Significance (ρ) was calculated according to Wilcoxon signed rank test. ρ = 1 indicated that both measurements were equal and that the method was robust against solar zenith angle.

Algorithm	Φ at High Solar Zenith Angle (28–43°)	Φ at Low Solar Zenith Angle (14–18°)	ρ
1	21.6 ± 4.1	24.2 ± 3.6	0.000
2	17.6 ± 4.1	17.8 ± 3.7	0.679
3	16.4 ± 4.3	17.1 ± 3.9	0.043
4	17.9 ± 4.3	18.0 ± 3.7	0.983

**Table 3:** Crown porosity measured in different olive varieties and dates for each pruning treatment. Values are mean ± standard deviation. Different letters showed significant differences (ρ < 0.05) according to Duncan’s test between different pruning treatments and in the same sampling date.

Variety	Sampling Date	Pruning Treatment	Φ (Algorithm 4) (%)
Hojiblanca	17/07/2013	Trunk-shaker-targeted	21.3 ± 0.9 a
		Canopy-shaker-targeted	23.8 ± 2.5 a
		Mechanical	13.8 ± 2 b
	05/06/2014	Trunk-shaker-targeted	13.9 ± 1.4 c
		Canopy-shaker-targeted	24.6 ± 3.8 a
		Mechanical	18.8 ± 3.9 b
Picual	15/07/2015	Trunk-shaker-targeted	18.9 ± 4.2 a
		Canopy-shaker-targeted	17.7 ± 3.4 a
		Mechanical	13.3 ± 1.5 b

**Table 4:** Pruning residues removed from the trees for each variety, treatment, and year. Values are mean ± standard deviation. Different letters in same columns showed significant differences (ρ < 0.05) according to Duncan’s test between different pruning treatments and in the same sampling date.

Variety	Pruning Treatment	Pruning Fresh Weight 2013 (kg·tree^−1^)	Pruning Fresh Weight 2014 (kg·tree^−1^)	Pruning Fresh Weight 2015 (kg·tree^−1^)	Pruning Cumulated Fresh Weight 2013–2014 (kg·tree^−1^)	Pruning Cumulated Fresh Weight 2013–2015 (kg·tree^−1^)
Hojiblanca	Trunk-shaker-targeted	39.5 ± 25.0 a	-	30.6 ± 18.9 a	39.5 ± 25.0 b	70.1 ± 35.4 b
	Canopy-shaker-targeted	20.5 ± 14.3 b	50.3 ± 32.2 a	32.8 ± 26.7 a	76.5 ± 31.1 a	108.7 ± 53.1 a
	Mechanical	-	55.5 ± 33.4 a	-	31.9 ± 14.8 b	55.5 ± 33.4 b
Picual	Trunk-shaker-targeted	47.9 ± 18.5 a	-	87.7 ± 38.1 a	47.9 ± 18.5 b	135.5 ± 47.5 a
	Canopy-shaker-targeted	34.1 ± 12.2 b	58.2 ± 24.6 a	31.9 ± 25.7 b	92.3 ± 25.6 a	124.2 ± 26.6 a
	Mechanical	-	36.8 ± 15.7 b	-	36.8 ± 15.6 b	36.8 ± 15.7 b

## Supplementary Materials

The following are available online at http://www.mdpi.com/1424-8220/16/5/723/s1. Figure S1: Pruning fresh weight relation with crown porosity; Table S1: Tree dimensions for tested years in different olive cultivars for each pruning treatment. Values are mean ± standard deviation. Different letters showed significant differences (ρ < 0.05) according to Duncan’s test between different pruning treatments and in the same year.

## Abbreviations

The following abbreviations are used in this manuscript:

PAR	Photosynthetically active radiation
Φ	Crown porosity
Ρ	Significance coefficient
r_Φ_	Repeatability calculated for a porosity value
σ	Variance
