# Supplementary material for: Olive Crown Porosity Measurement Based on Radiation Transmittance: An Assessment of Pruning Effect
Source: Sensors (Basel). 2016 May 19;16(5):723. doi: 10.3390/s16050723 (PMC4883414; doi:10.3390/s16050723)
Supplement: Supplementary file 1 [file sensors-16-00723-s001.pdf]

Supplementary Materials: Olive Crown Porosity Measurement Based on Radiation Transmittance: An Assessment of Pruning Effect

Francisco J. Castillo-Ruiz, Sergio Castro-Garcia, Gregorio L. Blanco-Roldán, Rafael R. Sola-Guirado and Jesús A. Gil-Ribes

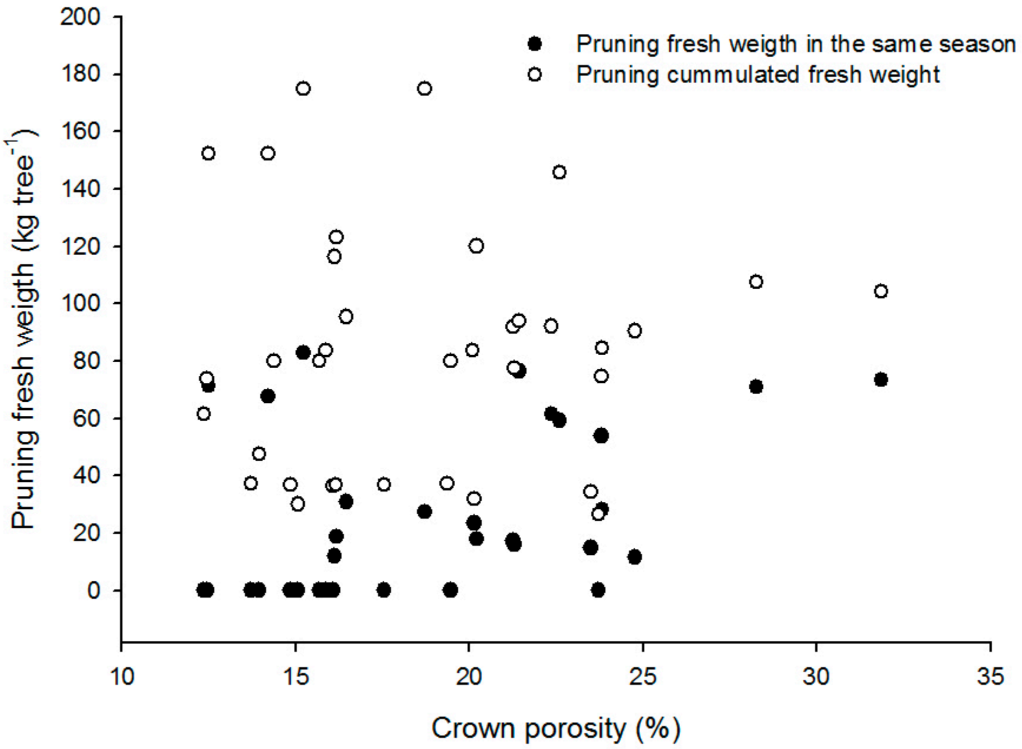

Figure S1. Pruning fresh weight relation with crown porosity.

Table S1. Tree dimensions for tested years in different olive cultivars for each pruning treatment. Values are mean ± standard deviation. Different letters showed significant differences (ρ < 0.05) according to Duncan's test between different pruning treatments and in the same year.

Variety	Year	Pruning Treatment	Skirt Height (m)	Tree Height (m)	Canopy Volume (m³)
Hojiblanca	2013 [1]	Trunk shaker targeted	0.7 ± 0.1 a	4.1 ± 0.3 a	92.2 ± 3.7 a
		Canopy shaker targeted	0.6 ± 0.1 a	4.1 ± 0.4 a	97.4 ± 20.3 a
		Mechanical	0.6 ± 0.1 a	3.6 ± 0.4 a	82.3 ± 2.3 a
	2014	Trunk shaker targeted	0.6 ± 0.2 a	3.9 ± 0.2 a	40.3 ± 9.2 a
		Canopy shaker targeted	0.5 ± 0.2 a	3.9 ± 0.3 a	42.7 ± 9.6 a
		Mechanical	0.4 ± 0.1 a	4 ± 0.5 a	67.5 ± 7.7 b
Picual	2015	Trunk shaker targeted	0.5 ± 0.1 b	3.9 ± 0.1 a	59.5 ± 11.8 a
		Canopy shaker targeted	0.4 ± 0.1 b	3.8 ± 0.3 a	63.7 ± 9 a
		Mechanical	0.3 ± 0.1 a	4.1 ± 0.1 b	72.5 ± 19.2 a

[1] In 2013, tree crown measurements were taken before to carry out pruning.
